# Autostent: a semi-automated approach to designing customized 3D-printed oral radiation stents for patients with head and neck cancer

**DOI:** 10.1186/s13014-025-02727-3

**Published:** 2025-11-17

**Authors:** Anshuman Agrawal, Rance B. Tino, Mohamed Zaid, Millicent Roach, Lianchun Xiao, Mark S. Chambers, Anna Lee, Eugene J. Koay

**Affiliations:** 1 Department of Radiation Oncology, The University of Texas MD Anderson Cancer Center, 1515 Holcombe Blvd, Unit 97, Houston, TX 77030 USA; 2 Division of VP-Research, Department of Biostatistics, The University of Texas MD Anderson Cancer Center, 7007 Bertner Avenue, Houston, TX 77030 USA; 3 Division of Surgery, Department of Head and Neck Surgery, The University of Texas MD Anderson Cancer Center, 1515 Holcombe Blvd, Houston, TX 77030 USA

**Keywords:** 3D printing, Stereolithography, Digital manufacturing, Head and neck cancer, Oral stent, Radiation therapy, Automation, Intraoral scanning, Software

## Abstract

**Background:**

Oral stents may reduce toxicities during radiation therapy for head and neck cancer (HNC). Customized 3D-printed oral stents offer faster production and achieve comparable patient-reported outcomes to conventionally fabricated stents. However, their design process remains time-consuming, lacks standardization, and relies heavily on skilled technicians. We hypothesized that semi-automating the design process for 3D-printed, mouth-opening, tongue-depressing (MOTD) stents could standardize the design workflow and decrease design time.

**Methods:**

Using oral stent design principles established over decades by oral oncologists, we created a customized computer program (Autostent) using MATLAB to semi-automate the design process of MOTD stents. We subsequently compared Autostent to a previously described method that utilized non-automated computer-aided design. Three users designed stents for four patients with HNC enrolled in a prospective observational study. These patients were selected based on their varying dental anatomies, and each user repeatedly designed an MOTD stent for each patient three times, employing both the non-automated and semi-automated methods. Both methods were compared in terms of design time and stent volume.

**Results:**

Semi-automation reduced the average design time by 23.6 min (51.2%, *p* = 0.001), regardless of user, dental anatomy, or trial number. Additionally, semi-automation decreased the average stent volume by 4.33 mL (12.9%, *p* = 0.016, univariate analysis). Although this reduction was not statistically significant when considering other experimental variables (*p* = 0.40, multivariate analysis), semi-automation did lower the variability in stent volume among users (the overall standard error of the mean decreased by 40%).

**Conclusion:**

Our semi-automated workflow for designing and fabricating customized, 3D-printed MOTD stents significantly improves efficiency and reduces variability in the design. While these results indicate greater consistency compared to manual methods, further development is warranted to achieve full automation and to optimize clinical integration.

**Supplementary Information:**

The online version contains supplementary material available at 10.1186/s13014-025-02727-3.

## Introduction

Radiation therapy (RT) effectively improves survival and local control in head and neck cancer (HNC), the fifth most common cancer in 2022, globally [1–3]. However, delivering higher radiation doses is offset by toxicities to organs-at-risk (OARs), particularly radiation-induced oral mucositis (RIOM) [4–6]. OARs are especially vulnerable to radiation exposure due to proximity to primary disease sites and their role in swallowing, speaking, and maintaining oral health. These toxicities can significantly diminish patients’ quality of life and result in treatment delays [6–8]. Toxicity-sparing devices such as oral positioning stents have been used for decades to enhance the therapeutic index of RT, targeting disease sites commonly involving the floor of the mouth, tongue, palate, and buccal mucosa [9–14].

In parallel with advances in RT, the development of patient-specific medical devices has evolved significantly with the integration of 3D printing, computer-aided design, and computer-aided manufacturing (CAD/CAM) technologies. These technologies have revolutionized the creation of customized medical devices across multiple disciplines, including dentistry, orthopedics, and maxillofacial surgery [15–17]. The advent of Artificial Intelligence (AI) has enabled high-throughput analysis of patient imaging data, showing potential for more accurate and efficient dental implantation planning [18]. Furthermore, CAD/CAM technologies in dentistry produce high-quality, standardized, accurate prosthetic restorations, demonstrating their feasibility for automating personalized devices [19]. Surgical guide fabrication has similarly benefited from automation, utilizing "static" template-based systems or "dynamic" surgical navigation technologies to transfer virtual treatment plans into actual patient treatments, particularly when using Virtual Surgical Planning (VSP) systems [20]. These approaches have shown promise in enhancing precision while reducing procedure time and improving clinical outcomes [19, 21]. Recently, combined approaches utilizing CAD/CAM, simulations, and complex algorithms have proven highly effective in realizing generative designs in maxillofacial applications [22].

Building on these advancements in customization and automation, similar principles are now being applied for developing oral positioning stents. Traditionally, fabricating fully customized oral positioning stents has required labor-intensive workflows with multiple in-person patient visits and collaboration between radiation oncologists and oral-maxillofacial surgeons [14, 23]. This process increases costs and limits the scalability of these devices in high-volume treatment centers, while leaving resource-limited centers without viable options for fully customized stents [23–32]. In response, 3D printing has garnered growing interest in oral oncology for its ability to produce highly customized oral positioning stents with faster turnaround times and lower costs. Additionally, HNC patients undergoing brachytherapy have benefited from 3D-printed molds or applicators, ensuring optimal placement and minimizing radiation exposure to OARs. [33, 34]

We previously demonstrated that our workflow for customized 3D-printed mouth-opening, tongue-depressing (MOTD) oral stents reduced fabrication time from 48 to 8 h without compromising patient outcomes [23, 28, 29]. This process uses an intraoral 3D scanner, CAD software, and a resin-based 3D printer. Despite the faster turnaround, the workflow remains time-consuming and relies heavily on user experience, making process standardization and streamlining difficult. While several commercial oral positioning stents are available, they are typically not patient-specific and lack semi-automated or automated digital workflows for customization. Currently, no automated solutions exist for designing customized oral positioning stents specifically for RT. To address this gap, we developed Autostent, a semi-automated in-house software that aims to reduce design time, standardize workflow, and improve stent consistency, potentially expanding access to customized oral stents for limited-resource cancer centers.

## Materials and methods

### Patient population

We evaluated four articulated stone models obtained from patients with base of tongue cancers, each presenting distinct dental anatomies (edentulous, hypodontic, normal occlusion, and class III malocclusion). All patients received definitive RT, with a mean total dose of 208 cGy (range: 200–212 cGy) delivered over 33 fractions (range: 30–35), between 2018 and 2019. They were referred to the oral oncologist for fabrication of standard MOTD stents and were enrolled under an Institutional Review Board-approved protocol (2017-0269) [23]. While other stent types (e.q, tongue-deviating, radiation carriers, etc.) are used depending on tumor location, this workflow focuses on the design and fabrication of MOTD stents. Workflows for other stent types may differ and are not covered in this study.

### Intraoral scanning of articulated stone models

We acquired a fabricated stone model for each patient and utilized a custom 3D-printed, 20 mm-tall bite block device to articulate each stone model and achieve a 20 mm interincisal distance between the maxillary and mandibular incisors. After removing the bite block device, we scanned the occlusal relationship using an intraoral scanner, resulting in a singular (.stl) model (TRIOS 3 Wired, 3Shape, Denmark). Subsequently, the resulting model was imported into MeshMixer software (Meshmixer V3.5.474, Autodesk Inc., USA) to separate the articulated maxillary and mandibular teeth models, followed by post-processing to convert them from surface mesh to ‘water-tight’ models, involving the filling of holes and removal of overlapping triangles. Finally, models were exported as two separate (.stl) files, the upper (maxillary) teeth model and the lower (mandibular) teeth model.

### Stent design

Three users were selected to independently complete three trials/repeats. In every trial, each user designed an MOTD stent for each of the four patients using two design approaches: (1) the original, non-automated approach in Meshmixer, and (2) the semi-automated approach in Autostent (see Tables S1 and S2 in Additional file 1 for a detailed comparison of the steps involved in both approaches). Additionally, the three users in this study covered the spectrum of stent-design experience: User 2 was a novice (one month with both approaches), User 1 was competent (six months with both approaches), and User 3 was an expert (over four years with approach 1; one month with approach 2).

**Fig. 1:**
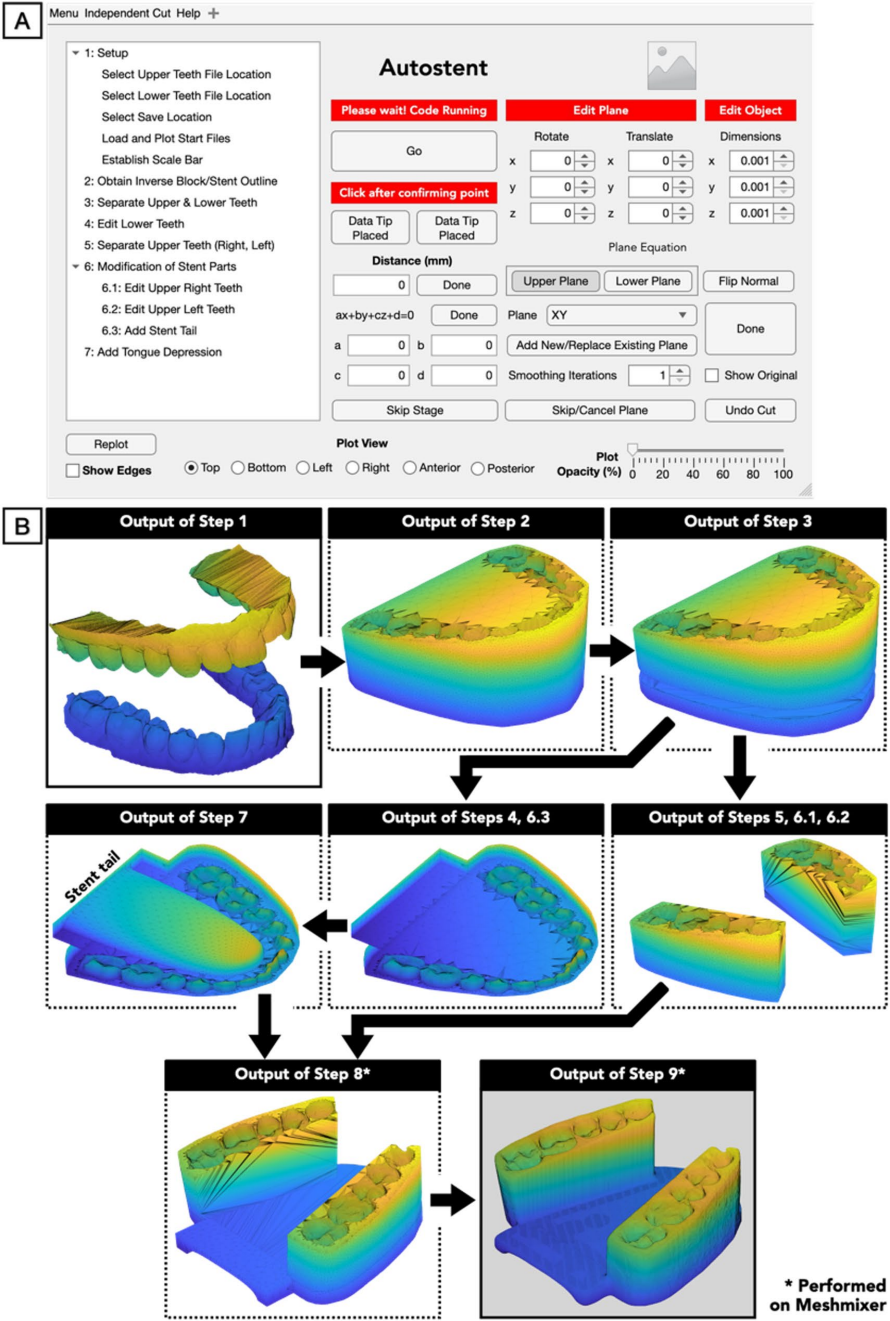
Autostent’s user interface (**A**) guides users through the workflow in a sequential manner from Steps 1 to 9. Outputs of each step of the semi-automated workflow (**B**). Steps 1–7 are completed on Autostent (listed on the left in **A**). Steps 8–9 are completed on Meshmixer

*Semi-automated method* The design workflow was semi-automated using MATLAB R2020a (Mathworks Inc., USA) (see Table 1 and Fig. 1 for an overview of Autostent’s workflow). MATLAB’s Application Designer was utilized to create the user interface for Autostent (see Fig. 1A), and the workflow was standardized into nine steps (see Fig. 1B, which visualizes each step’s output).

**Table 1:** Semi-automated design workflow using Autostent and Meshmixer V3.5.474 (Autodesk Inc., USA)

Software	No^a^	Tasks	User input required?	Description
Autostent	1	Setup	Yes	a. The patient’s 3D intraoral scans of the maxilla and mandible are imported, and a 20 mm inter-incisal opening is used to define a scale bar
	2	Obtain Inverse Block/Stent Outline	No (Automated)	b. Best-fit planes for the maxilla and mandible are created using principal component analysis c. The maxillary plane is located at 60% of the height (from the upper gums), while the mandibular plane is at 40% (from the lower gums) d. The user can adjust these planes. The combined convex hull of the patient’s maxillary and mandibular structures is calculated, expanded outward by 1.5%, and sliced off at the defined planes e. The patient’s teeth are removed from the sliced convex hull to create a customized dental impression [35]. f. The slicing of the 3D mesh is accomplished using Constructive Solid Geometry functions in iso2mesh toolbox g. Every plane cut is executed as a Boolean subtraction between the mesh and a cube, with one face angled to align with the plane
	3	Separate Upper & Lower Teeth	No (Automated)	h. The upper and lower teeth are separated by a plane set 2 mm above the lower central incisor (identified in step 1 while defining the scale bar), with its slope based on the mandibular plane defined in step 2
	4	Edit Lower Teeth	Yes ^b^	i. The third molars are removed from the lower stent
	5	Separate Upper Teeth (Right, Left)	No (Automated)	j. The left and right upper tooth supports are created by modifying the upper stent with three predefined, user-customizable vertical planes for each side. The canines, both premolars, and the first two molars are included. Although unnecessary, the supports can each be further modified in steps 6.1–6.2
	6	Modifications of Stent Parts	Yes^b^	
	6.1	Edit Upper Right Teeth	Optional	
	6.2	Edit Upper Left Teeth	Optional	
	6.3	Add Stent Tail	Yes^b^	k. The stent tail needed to depress the tongue is created by combining the lower stent with a user-customizable box extending 10 mm beyond the second molar
	7	Add Tongue Depression	Yes^c^	l. A standard tongue-like mesh is placed manually and subtracted from the lower stent to create space for the patient’s tongue. The mesh is placed such that a) there isn’t too much space between the tongue and the edges of the lower teeth, and b) the plate thickness is > 10 mm
Meshmixer	8	Boolean addition	Yes^d^	m. The upper left and right stent supports, along with the lower stent, are combined
	9	Smoothing	Yes^e^	n. Sharp edges are smoothed, and the file is exported for 3D printing

^a^After each step, Autostent saves the resulting triangulated mesh as a stereolithography (.stl) file to a user-defined folder to prevent data loss. These are used to complete steps 8–9 in Meshmixer

^b^While user input is required, preset planes are presented to speed-up the step. The step wasn’t fully automated due to challenges in automatically identifying the appropriate boundaries of the specific teeth involved

^c^The tongue mesh was a fully customizable ellipsoid-like mesh whose dimensions and position could be modified along all three axes by the user. To facilitate faster user-positioning, it is initially auto-placed at the center of the lower teeth and translated posteriorly by 17.5 mm so that the tongue’s tip is behind the incisors. This step wasn’t fully automated due to challenges in automatically identifying the boundaries of the lower teeth to auto-size the tongue mesh to fit between them

^d^This step required user input since it needed to be performed in Meshmixer. We had attempted to perform this step automatically on Autostent but ultimately chose Meshmixer due to its faster and more robust Boolean Addition tool

^e^This step required user input because it was challenging to auto-identify the sharp edges on the stent that were not from the dental impression. We had attempted automatically smoothing all edges with faces that formed a certain “sharp” angle, but this changed the dental impression. To prevent his undesirable consequence, we ultimately chose to perform this step manually on Meshmixer

### Stent fabrication

Stent models were imported into a slicer (PreForm v3.33.1, Formlabs Inc., USA) and oriented with the anterior region facing the build platform, minimizing support structures and preventing supports from forming on the impression regions. Subsequently, stents were fabricated using an autoclavable, biocompatible photopolymer resin (Surgical Guide Resin, Formlabs Inc., USA) on a Form 2 3D printer (Formlabs Inc., USA), with a slice thickness of 0.1 mm. After printing, support structures were removed from the stents by hand, followed by a 20 min washing with a 99% isopropyl alcohol bath (Form Wash, Formlabs Inc., USA), drying with paper towels, and ultra-violet (UV) curing for 30 min (Form Cure, Formlabs Inc., USA). Finally, stents underwent the standardized post-processing procedures for cleaning and polishing conventional oral stents using polishing drill bits, a lathe machine, and a rag wheel.

### Stent evaluation

The stent design time was recorded, and the stent volumes across the three trials were measured to assess inter-user variability. Furthermore, linear mixed models that included patient dental anatomy as random effects were fitted to assess whether the differences in time and volume between the two methods (per user, per trial, for each patient) were statistically significant (*p* < 0.05). To account for the effects of users and trials, this analysis was repeated after adding users and trials as covariates (multivariate analysis). ANOVA and two-sample t-tests were employed to compare the volumes between the two methods for each user.

## Results

### Design time

**Fig. 2:**
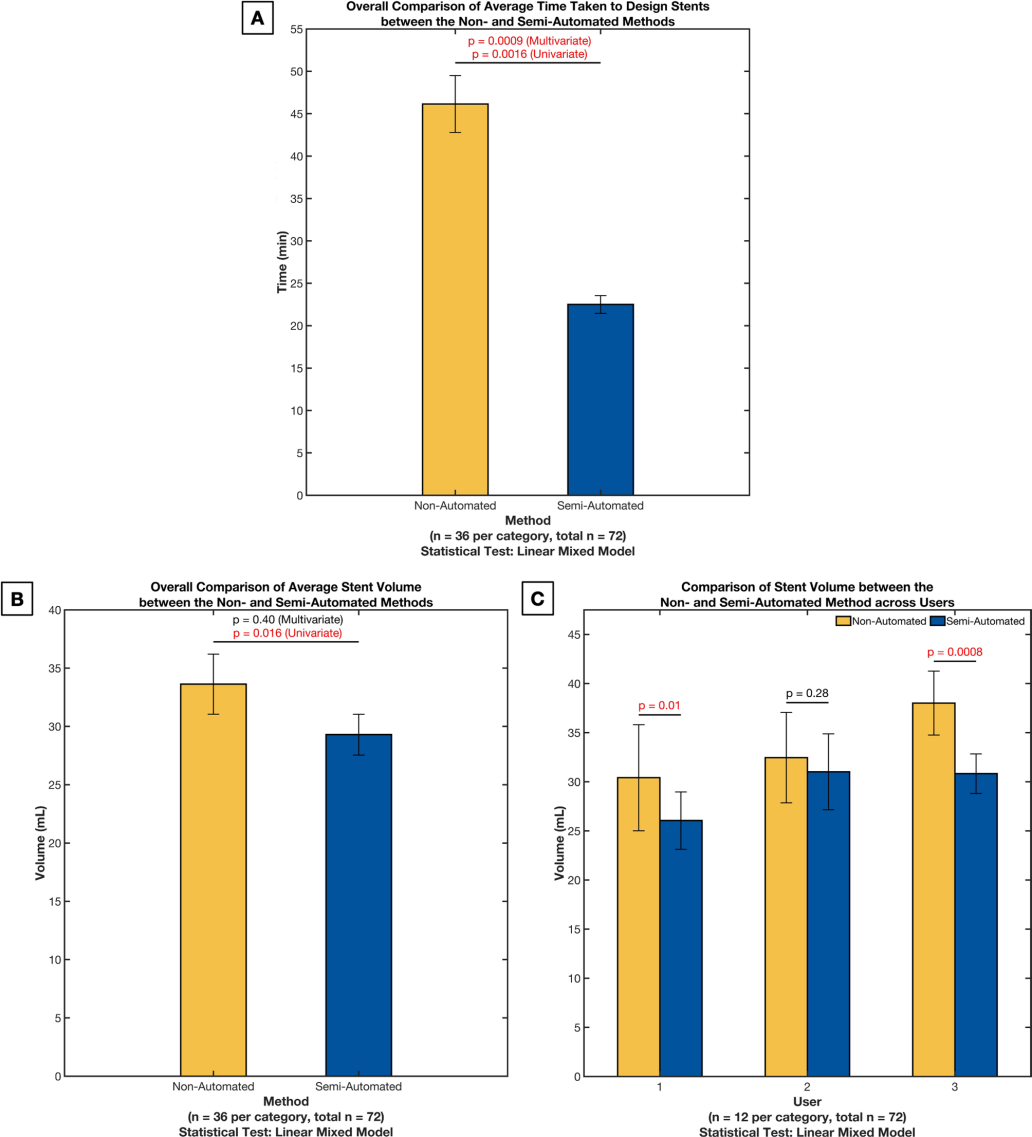
Results comparing the non-automated and semi-automated methods using the design time (**A**), stent volume (**B**), and stent volume across users (**C**). The error bars represent the 95% confidence interval for the mean

The analysis revealed that designing stents was considerably faster with Autostent (mean difference = 23.6 min, 95% CI 17.6, 29.7, *p* = 0.0016, Fig. 2A). Even after adjusting for the effects of users and trials, the difference remained statistically significant (*p* = 0.0009).

### Stent volume

**Fig. 3:**
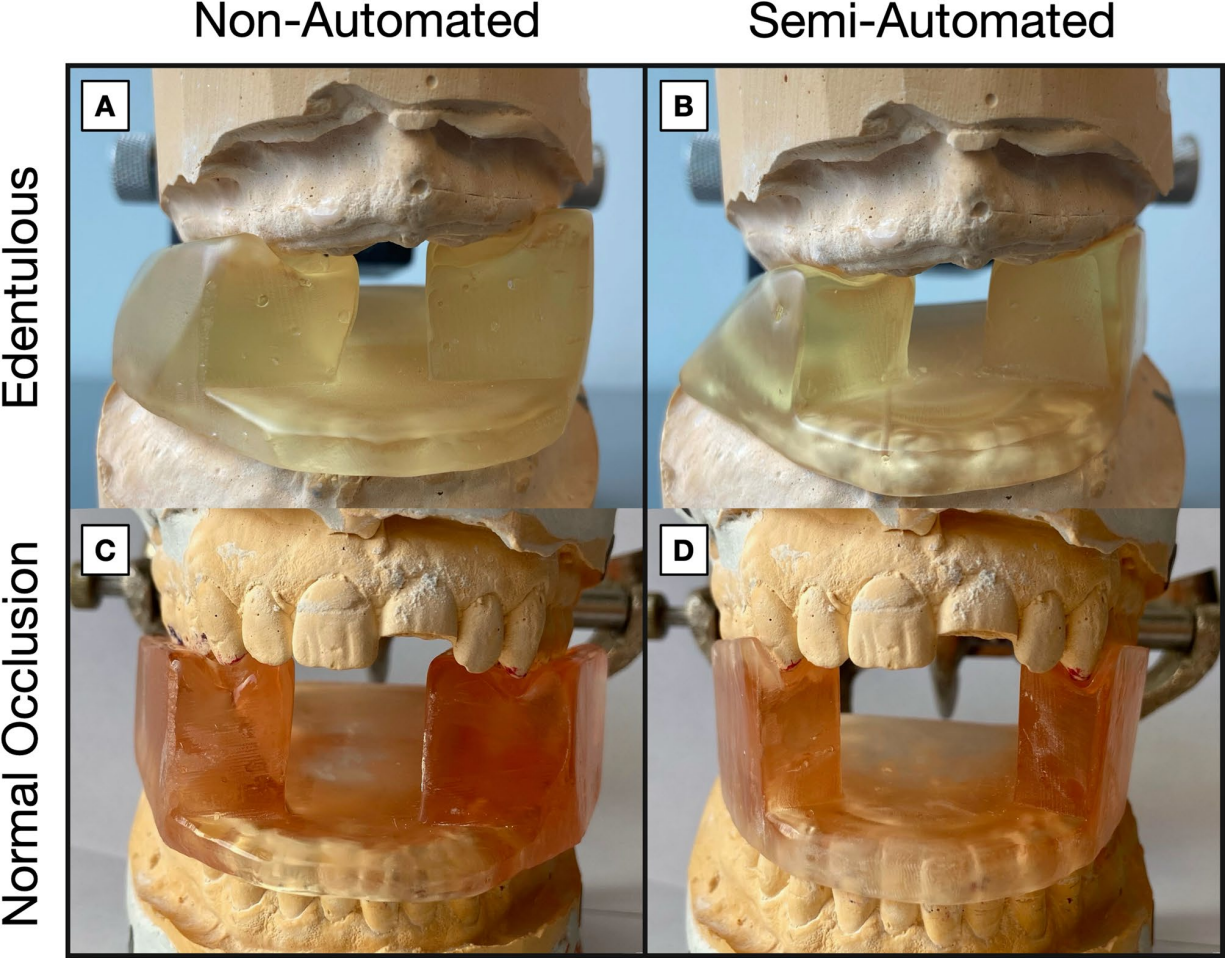
3D printed stents designed using the non-automated (**A**, **C**) and semi-automated (**B**, **D**) method for the edentulous (**A**, **B**) and normal occlusion (**C**, **D**) dental anatomies. The boundary of the stent designed using the semi-automated method is more compact, thus reducing the volume and potentially resulting in a more comfortable fit

The univariate analysis showed that stents designed using Autostent had significantly lower volumes (mean difference = 4.33 mL, 95% CI 2.20, 6.47, *p* = 0.016) (see Figs. 2B and 3). After adjusting for the effects of users (*p* < 0.0001) and trials (*p* = 0.75), the difference was no longer statistically significant (*p* = 0.40). Analyzing the stent volumes between the two methods for each user (Fig. 2C) revealed that semi-automation led to lower variability for all users (the standard error of the mean decreased by 1.1, 0.3, and 0.6 for users 1, 2, and 3, respectively, and by 0.4 overall).

## Discussion

In this study, we developed a semi-automated digital workflow involving an in-house MATLAB tool, Autostent, to design and fabricate fully customized MOTD stents. Our results demonstrate measurable improvements over non-automated methods, most notably a 23 min reduction (51.2%) in design time and a 40% reduction in the variability of stent volumes across users. These findings highlight the potential of the Autostent tool to streamline the stent design process and improve consistency in clinical practice. Although we did not directly measure standardization as an outcome variable, the step-by-step procedure integrated into the Autostent tool establishes a consistent design process, as reflected by the observed reduction in variability of stent volumes. To the best of our knowledge, no other semi-automated or automated software solutions are specifically designed for the rapid and streamlined creation of customized oral positioning stents in the context of HNC-RT. While related technologies have been described for other dental and maxillofacial applications, our approach represents a new contribution in this field by directly addressing the unique clinical and logistical challenges associated with oral stent fabrication for HNC-RT applications. With the increased use of oral stents among patients with HNC undergoing RT, these advancements represent an important step toward enabling broader clinical implementation and ensuring greater access to fully customized oral stents. [36]

The clinical challenge of treating patients with HNC using high-dose radiation while minimizing RIOM may be partially addressed by using customized oral positioning stents that help improve the therapeutic index [9–13]. As personalized RT continues to advance, the adoption of patient-specific devices designed to spare OARs, especially in anatomically complex regions, has become increasingly prevalent. Comparable strategies have been implemented in brachytherapy, where customized ‘spacers’ are used to physically displace OARs, and have shown encouraging results in improving dose conformity and minimizing treatment-related toxicity [34]. Standardizing the design and customization process through semi-automated methods, such as the approach introduced in this study, has the potential to streamline the integration of these devices into routine clinical practice, making personalized treatment more accessible and consistent.

Recent studies have shown that CAD and filament-based 3D printing can be used to design tongue-depressing stents, facilitating the attachment of dental silicone for teeth impressions and placement [24–26, 32]. Cleland et al. showed that combining molding silicone with a simple modular stent design (Autodesk, Mill Valley, US) can accommodate variations in patient oral anatomy, effectively displacing the tongue and buccal mucosa, thereby reducing the potential for dose-related toxicities [24, 25]. By adopting a fully digital approach similar to our study, Bruno et al. demonstrated the clinical potential of combining an intraoral 3D scanner, dental-based software (Ceramill^®^; Amann Girrbach AG), and resin-based 3D printing to fabricate a tongue-depressing stent. [31]

While our study yielded results comparable to existing studies, direct comparison is challenging due to variability in reported variables, with some studies providing different measurements and others lacking quantitative assessments. Our analysis was necessarily limited by four patient cases and three users, curtailing the statistical power of the multivariate stent volume analysis (Fig. 2B). Even so, identical design instructions (Tables S1 and S2, Additional file 1) did not eliminate user‑specific choices (such as approximating the appropriate stent tail length, the distance from the teeth to make cuts, etc.), and those choices are reflected in the differing average stent volumes observed in Fig. 2C. Because volume is the cumulative result of several modeling operations, identifying a single decisive step is difficult. Nevertheless, careful inspection of the final mesh models generated by each workflow (examples shown in Fig. 3) indicates three stages where user discretion most plausibly drives the observed differences. First, shell construction differs fundamentally between the two approaches. The semi-automated approach wraps the dentition with a convex-hull expansion whose margin is set in code, so every user, and indeed every patient, receives a similar shell. The non-automated approach requires the user to approximate that hull using a combination of flat cutting planes, which, when combined with the need to be time-efficient and not create a shell that is too close to the patient’s teeth, likely produces a thicker, less uniform wall. Second, the users manually establish the plane separating the maxillary and mandibular teeth models, which defines the thickness of the plate that depresses the tongue. Positioning this plane a few millimeters higher or lower alters the entire plate thickness and appreciably increases the stent volume. In the non-automated method, the plane is entirely dependent on the user, predictably leading to higher variability. Even in the semi-automated method, where the plane is set at a pre-determined distance above the tip of the lower central incisor, the angle of the plane could have been adjusted by the user in a previous step, thus leading to some inter-user variability in the stent volume. Third, while users were given a few design recommendations, the tongue mesh is primarily scaled and positioned by eye. Users who spend extra time scaling the mesh and positioning it to achieve a closer fit with the neighboring lower teeth would likely create stents with a lower volume. In contrast, coarser placement could lead to a higher stent volume. Due to the lack of intermediate mesh models in the non-automated approach, these inferences stem from user experience while designing and comparing completed mesh models. The latter two user‑sensitive steps (steps 8 and 9, Table 1) could, in principle, be standardized if the software could automatically segment individual teeth and determine the occlusal plane based on predefined relationships to these teeth. Recent gains in AI-based 3D dental segmentation make such automation increasingly feasible; a future Autostent release that embeds those algorithms would convert today’s subjective judgments into fixed parameters and, by extension, reduce the variabilities seen in Fig. 2C. Expanding the patient cohort and enlisting users with a wider range of experience will be equally important to test whether a semi- or fully-automated pipeline can deliver consistent results across diverse anatomical scenarios.

Future work will focus on integrating computational algorithms to automatically identify dental landmarks and fully automate the design process, eliminating the need for third-party CAD software by completing all steps within Autostent. A revised version of the semi-automated workflow is currently being evaluated in a Phase II randomized trial (protocol 2020-1153) to further assess Autostent’s clinical potential in developing both tongue-depressing and tongue-lateralizing stents.^30^

## Conclusion

In summary, our semi-automated digital workflow represents an advancement in the preparation of fully customized MOTD stents for HNC radiation therapy. By substantially reducing design time by over 50% and decreasing variability in stent volume across users by 40%, this approach addresses key barriers to broader clinical adoption of patient-specific oral positioning stents. Beyond improving efficiency, these enhancements have the potential to lower costs, expand access, and contribute to better patient outcomes through full customizability and reliable device fabrication. Moving forward, integrating more complex computational algorithms to fully automate stent designs and the implementation of multi-center, randomized clinical trials will be pivotal in validating and maximizing the clinical impact of this technology.

## Supplementary Information

Below is the link to the electronic supplementary material.

Supplementary Material 1.

## Data Availability

All data generated and analyzed in the study have been included in the manuscript. Other data for the study can be requested from the corresponding authors and shared after approval from the Institutional Review Board.

## Abbreviations

3D: 3-Dimensional
ANOVA: Analysis of variance
AM: Additive manufacturing
CAD: Computer-aided design
CAM: Computer-aided manufacturing
HNC: Head and neck cancer
MOTD: Mouth-opening, tongue-depressing
RIOM: Radiation-induced oral mucositis
RT: Radiation therapy
