# Supplementary material for: Autostent: a semi-automated approach to designing customized 3D-printed oral radiation stents for patients with head and neck cancer
Source: Radiat Oncol. 2025 Nov 17;20:170. doi: 10.1186/s13014-025-02727-3 (PMC12625475; doi:10.1186/s13014-025-02727-3)
Supplement: Supplementary file 1 — Supplementary Material 1. [file 13014_2025_2727_MOESM1_ESM.docx]

**Additional File 1: Abridged Stent Design Standard Operating Procedure**

Detailed instructions for stent creation using the non-automated and semi-automated design methods are presented in Tables S1 and S2, respectively.

**Table S1. Steps for the non-automated stent design approach using Meshmixer V3.5.474 (Autodesk Inc., USA).**

| **Step #** | **Description** |
| --- | --- |
| **1** | 1. Upload/Load teeth scan models to MeshMixer. |
|  | 1. Combine upper and lower teeth models. |
|  | 1. Upload/Load impression block model. MeshMixer will automatically reposition this model in the workspace. |
|  | 1. To prevent loss of data due to errors, save the MeshMixer file as a ‘.mix’ file (XX_c3_t1.mix, for Case 3, Trial 1 by user XX). |
| **2** | 1. Position the impression block such that it encapsulates much of the teeth scan (no need to rotate the block to align to the teeth as the next plane cut can do that. |
|  | 1. Boolean Subtract the teeth models from the impression block model. |
| **3** | 1. Generate an upper teeth plane that cuts through ≈50% of the depth of the upper teeth. The canines to the 2^nd^ molars are important here (cut type: “Discard”). 2. Generate a lower teeth plane that cuts through ≈50% of the depth of the lower teeth. All lower teeth until the 2^nd^ molar are important here (cut type: “Discard”). 3. Clear the extra block in front of the front teeth by generating a plane to cut from the lower incisor to the upper incisor) (cut type: “Discard”). 4. Clean the boundary of the stent using the same plane cutting method. Keep upper and lower teeth into account when doing this (cut type: “Discard”). 5. Perform two inner plane cuts next to the right and left upper teeth model, which generate the upper teeth supports (cut type: “Keep both” 6. Cut behind the 2^nd^ molar of the upper and lower teeth models using the plane cutting process (cut type: “Keep both”) 7. Separate the upper and lower teeth models using the plane cutting process (cut type: “Keep booth”) |
| **4** | 1. Select and delete unnecessary regions of the stent. |
|  | 1. Perform additional plane cuts to refine the boundary of the stent (cut type: “Discard”). |
|  | 1. Ensure the stent model is ‘watertight’ by clicking the “Make Solid” tool (Solid accuracy: 325, Mesh density: 325). Then click ‘Update’. |
| **5** | Generate the tongue impression on the stent model by manually ‘painting’ regions of tongue interface, then use the ‘Transform’ tool to create the concaved, tongue impression. |
| **6** | Under the “Sculpt” tool, use the “RobustSmooth” brush to refine and smooth the tongue impression and the teeth impression regions to ensure that no sharp edges exist. |
| **7** | Export (.stl) model. |

**Table S2. Steps for the semi-automated stent design approach using Autostent and Meshmixer V3.5.474 (Autodesk Inc., USA).**

| **Step #** | **Description** |
| --- | --- |
| **1** | 1. Select the upper/maxillary teeth (.stl) file. |
|  | 1. Select the lower/mandibular teeth (.stl) file. |
|  | 1. Select save location/directory. |
|  | 1. Load and plot the imported upper and lower teeth files. |
|  | 1. Establish scale bar. |
| **2** | Obtain the inverse block/stent outline. |
| **3** | Separate the upper and lower teeth models. |
| **4** | Edit lower teeth model. |
| **5** | Separate the upper teeth into left and right sub parts. |
| **6** | 1. Edit the upper right teeth model. |
|  | 1. Edit the upper left teeth model. |
|  | 1. Edit stent tail model. |
| **7** | Add tongue depression. |
| **8*** | Import the three parts generated by Autostent to MeshMixer and combine them using the Boolean addition tool. Subsequently, make the resulting boolean model ‘watertight’ using “Make solid” tool (Solid accuracy: 325, Mesh density: 325). Then click “Update”. |
| **9*** | Under the “Sculpt” tool, use the “RobustSmooth” brush to refine and smooth the tongue impression and the teeth impression regions to ensure that no sharp edges exist. |

******* *Performed on Meshmixer.*
